# Assessment of hepatitis c core antigen in epithelial salivary gland neoplasms (ex-vivo study)

**DOI:** 10.1186/s12903-024-04632-9

**Published:** 2024-08-06

**Authors:** Hadeel Ahmad Kotat, Awatef Ibrahim Draz, Marwa Mokbel ElShafei, Hatem Wael Amer

**Affiliations:** 1 Oral Pathology Department, Faculty of Oral and Dental Medicine, Misr International University, Cairo, Egypt; 2 Oral and Maxillofacial Pathology Department, Faculty of Dentistry, Cairo University, Cairo, Egypt

**Keywords:** Hepatitis C, Salivary gland neoplasms, HCV core antigen, Immunohistochemistry

## Abstract

**Background:**

Salivary gland neoplasms (SGNs) pose a challenge to both pathologists and clinicians. Despite research, the etiology of these neoplasms remains unclear. This study aimed to identify any potential association between the presence of hepatitis C virus (HCV) at the protein or gene level and epithelial salivary gland neoplasms.

**Methods:**

Formalin-fixed paraffin-embedded (FFPE) blocks of epithelial salivary gland neoplasms were retrieved from the archives of the Oral and Maxillofacial Pathology Department, Faculty of Dentistry, Cairo University within the 5-year period from 2016 to 2020. Immunohistochemistry was used to assess HCV core antigen, while reverse transcription polymerase chain reaction was employed for the evaluation of HCV RNA.

**Results:**

A total of 44 specimens were collected, 28 of which were benign neoplasms and 16 were malignant neoplasms. There was a statistically significant difference in HCV positivity between the two groups (*P*-value = 0.036). Benign tumors showed a statistically significant lower percentage of positive cases than malignant tumors. The localization of staining was also evaluated, revealing various patterns of HCV core antigen expression, including diffuse cytoplasmic, patchy cytoplasmic, nuclear, and a combination of nuclear and cytoplasmic expression. There was no statistically significant difference between the expression patterns in benign and malignant tumors (*P*-value = 0.616). Given that Pleomorphic Adenoma and Mucoepidermoid Carcinoma were the predominant tumor types in this study, four cases were selected for RNA detection. HCV RNA was detected in all cases using RT-PCR.

**Conclusions:**

HCV core antigen is frequently detected in SGNs and is suggested to be a potential risk factor for the development of these neoplasms. Further studies are required to discover other biomarkers, their roles, and the pathways associated with HCV in SGNs.

## Background

Salivary gland neoplasms (SGNs) pose a major challenge to both pathologists and clinicians. However, the etiology of these neoplasms remains unclear [1]. Inflammation is considered the seventh hallmark of neoplasia. The presence of chronic inflammation accompanied by an infectious agent may accelerate neoplastic transformation [2].

Hepatitis C virus (HCV) is an enveloped single-stranded positive-sense RNA virus [3]. In Egypt, this virus is a public health concern. Faced with this major health and economic burden, Egypt established its first national control program for HCV in 2008, aimed at expanding access to treatment. By 2014, Egypt had issued its second national program for eliminating HCV, prioritizing prevention, education, and improved patient care for individuals affected by the virus [4]. The HCV epidemic in Egypt can be traced back to mass transmission through inadequately sterilized glass syringes and needles used in campaigns by the Egyptian Ministry of Health to treat rural endemic schistosomiasis. The ongoing transmission of HCV in Egypt is primarily linked to medical and dental practices [5]. The national treatment strategy for the control of HCV infection in Egypt was set by the National Committee for Control of Viral Hepatitis, and the Egyptian government has taken positive steps to control HCV as a public health threat, aiming to eliminate the disease by the year 2030 [6]. The World Health Organization (WHO) has formulated a global health sector strategy for viral hepatitis, targeting the elimination of HCV as a public health threat by 2030 [7].

Oral extrahepatic manifestations in individuals with chronic hepatitis C often include salivary gland disorders, highlighting HCV as a sialotropic virus. HCV antigens have been detected in salivary gland epithelial cells and in the saliva of patients with chronic sialadenitis and non-Hodgkin’s lymphoma [8].

Several studies have suggested that HCV may have oncogenic effects. Tumors predominantly associated with HCV include Hepatocellular Carcinoma, non-Hodgkin Lymphoma, Intrahepatic Cholangiocarcinoma, and pancreatic cancer. Recently, HCV has been highlighted as a possible risk factor for the development of head and neck cancer [9, 10]. HCV may promote carcinogenesis directly or indirectly by promoting a proinflammatory state that is favorable for cancer development [11]. Nevertheless, the specific mechanisms linking HCV to head and neck neoplasms remain unclear and require further investigation [3, 12]. Hence, the main aim of this study was to assess the possible association between hepatitis C virus and epithelial SGNs.

## Methods

### Samples

This was an ex vivo study on formalin-fixed paraffin-embedded (FFPE) tissue specimens retrieved from the archives of the Oral and Maxillofacial Pathology Department, Faculty of Dentistry, Cairo University. Inclusion criteria included cases of epithelial SGNs diagnosed within 5 years (2016–2020), all age groups, and both genders. The blocks were examined again to confirm the diagnosis and specimens with optimal tissue adequacy were selected. All the cases were reviewed according to the WHO (2022) classification of head and neck tumors. Data regarding age, gender, tumor site, and diagnosis were collected and recorded from the histopathological reports. If reported, HCV seropositivity data were also collected and matched with their results.

### Staining

**Fig. 1:**
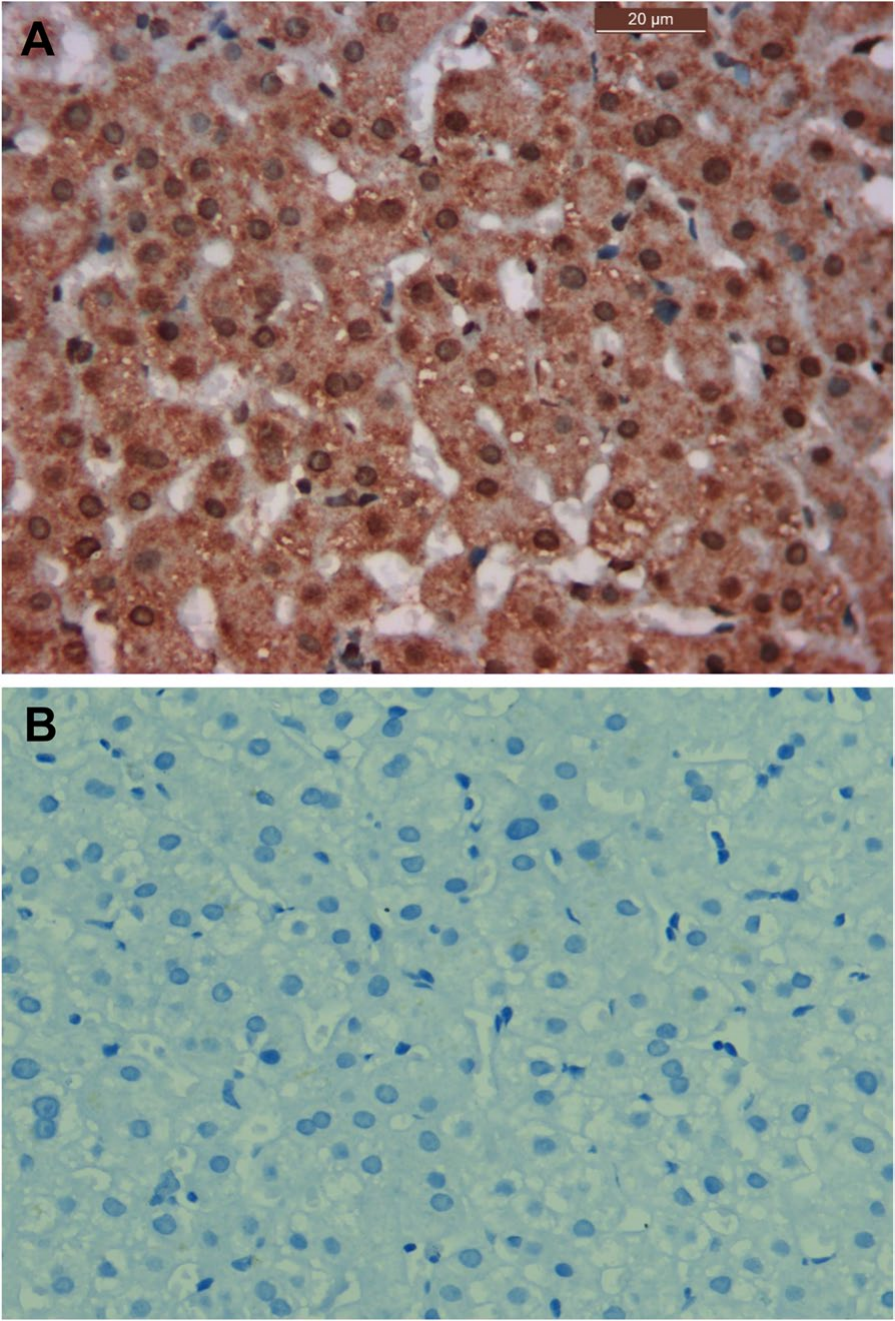
Photomicrograph of the positive control showing strong diffuse staining in hepatocytes of liver tissue sample from a chronically infected HCV patient, magnification 400x (**A**). Photomicrograph of the negative control showing negative expression in hepatocytes of liver tissue sample from a chronically infected HCV patient following omission of the primary antibody, magnification 400x (**B**)

The specimens were sectioned into 4-µm thick slices. These specimens were analyzed for the presence of HCV core antigen by immunohistochemistry. A staining kit (Ventana Medical Systems, Tucson, AZ, USA) was used according to the instructions provided to evaluate the expression of HCV core antigen. Commercially available concentrated polyclonal rabbit anti-HCV core antigen was used for this purpose (catalog #YPA14167F, 7F, Bldg. B, High-tech Venture Park, #107 Erlang Chuangye Rd, Jiulongpo District, Chongqing 400039, China). In every run, positive and negative controls were included to determine antigenic expression patterns (Fig. 1).

### HCV RNA detection

Reverse transcription polymerase chain reaction (RT-PCR) was performed at Science Way Laboratory in Nasr City, Cairo, Egypt, involving several detailed steps. First, small sections of paraffin-embedded tissue underwent deparaffinization using xylene and ethanol, followed by RNA extraction through lysis and proteinase K treatment, with subsequent washing and centrifugation steps. The RNA was then transformed into complementary DNA (cDNA) using specific reagents and PCR machine settings, including initial denaturation, reverse transcription, enzyme inactivation, and cooling. For PCR amplification, outer primers facilitated initial cDNA synthesis, while nested primers targeted the 5′UTR of the HCV gene, with results visualized on a 2% agarose gel stained with ethidium bromide.

### Evaluation

Two methods were employed to evaluate the stained sections: positive immunoreaction and localization of the immunostain within the tissues, which were examined using a transmitted light microscope. The criteria for identifying antigen-positive areas included staining of the nucleus, cytoplasm, or both the nucleus and cytoplasm of the tumor cells. All immunostained sections were analyzed using a computer image analyzer system equipped with ImageView software at the Analytica Research Center in Giza, Egypt. The area percentage of cells displaying HCV core antigen immunostaining was measured using ImageJ software at × 400 magnification by light microscopy and displayed on the monitor screen.

### Data analysis

Numerical data were explored for normality by checking the distribution of data and using tests of normality (Kolmogorov-Smirnov and Shapiro-Wilk tests). Age data showed a normal (parametric) distribution, whereas area % data showed a non-normal (non-parametric) distribution. Data were presented mean, standard deviation (SD), median and range values. For parametric data, Student’s t-test was used to compare the mean age values between the two groups. For non-parametric data, Mann-Whitney U-test was used to compare the two groups. Qualitative data were presented as frequencies and percentages. Chi-square test and Fisher’s Exact test were used for comparisons of qualitative data. The significance level was set at *P* ≤ 0.05. Statistical analysis was performed using IBM SPSS Statistics for Windows, Version 23.0. Armonk, NY: IBM Corp.

## Results

In this study, 44 cases of epithelial SGNs were examined. Among these cases, 28 were classified as benign, whereas the remaining 16 were malignant. The benign tumors included 21 Pleomorphic Adenomas, 4 Basal Cell Adenomas, 2 Canalicular Adenomas, and 1 Papillary Oncocytic Cystadenoma. The malignant tumors included 10 Mucoepidermoid Carcinomas, 2 Adenoid Cystic Carcinomas, 2 Myoepithelial Carcinomas, and 2 Oncocytic Carcinomas. The clinical data are summarized in Tables 1 & 2.

**Table 1:** Descriptive statistics and results of Chi-square test and Student’s t-test for comparisons between demographic data in the two groups

Demographic data	Benign (*n* = 28)	Malignant (*n* = 16)	*P*-value
Gender [n, (%)]			
Male	10 (35.7%)	9 (56.3%)	0.186
Female	18 (64.3%)	7 (43.8%)	
Age [Mean, SD]	46 (13.9)	34.3 (14.9)	0.013*

^*^Significant at *P* ≤ 0.05

**Table 2:** Frequencies (n), percentages (%) and results of Fisher’s Exact test for comparison between tumor site in the two groups

Site	Benign (*n* = 28)		Malignant (*n* = 16)		*P*-value	*Effect size (v)*
	n	%	n	%		
Alveolar ridge	2	7.1	0	0	0.036*	0.629
Hard palate	15	53.6	6	37.5		
Junction between hard and soft palate	3	10.7	2	12.5		
Labial mucosa	0	0	2	12.5		
Lower anterior mucosa	0	0	2	12.5		
Lower posterior gingiva	1	3.6	0	0		
Maxillary tuberosity	1	3.6	0	0		
Retromolar area	0	0	3	18.8		
Soft palate	2	7.1	1	6.3		
Upper posterior area	1	3.6	0	0		
Upper lip	3	10.7	0	0		

^*^Significant at *P* ≤ 0.05

### HCV core antigen expression

In this study, 33 cases of the 44 cases examined, accounting for 75% of the entire sample, expressed positivity for HCV core antigen immunostaining (Table 3).

**Table 3:** Frequencies (n), percentages (%), and results of Fisher’s Exact test for comparisons between HCV positivity in the two groups

HCV positivity	Benign (*n* = 28)		Malignant (*n* = 16)		*P*-value	*Effect size (OR)*
	n	%	n	%		
Positive	18	64.3	15	93.8	0.036*	8.333
Negative	10	35.7	1	6.3		

*OR* Odds Ratio

^*^Significant at *P* ≤ 0.05

The analysis involved comparison of anti-HCV core antigen expression across various SGNs. Among the positive cases in the benign group, 15 of the 21 Pleomorphic Adenomas and 3 of the 4 Basal Cell Adenomas exhibited positive expression for anti-HCV core antigen.

When examining the expression of anti-HCV core antigen within malignant SGNs, it was observed that all cases of Mucoepidermoid Carcinoma, Myoepithelial Carcinoma, Oncocytic Carcinoma, and one of the two cases of Adenoid Cystic Carcinoma exhibited positive expression of anti-HCV core antigen.

**Fig. 2:**
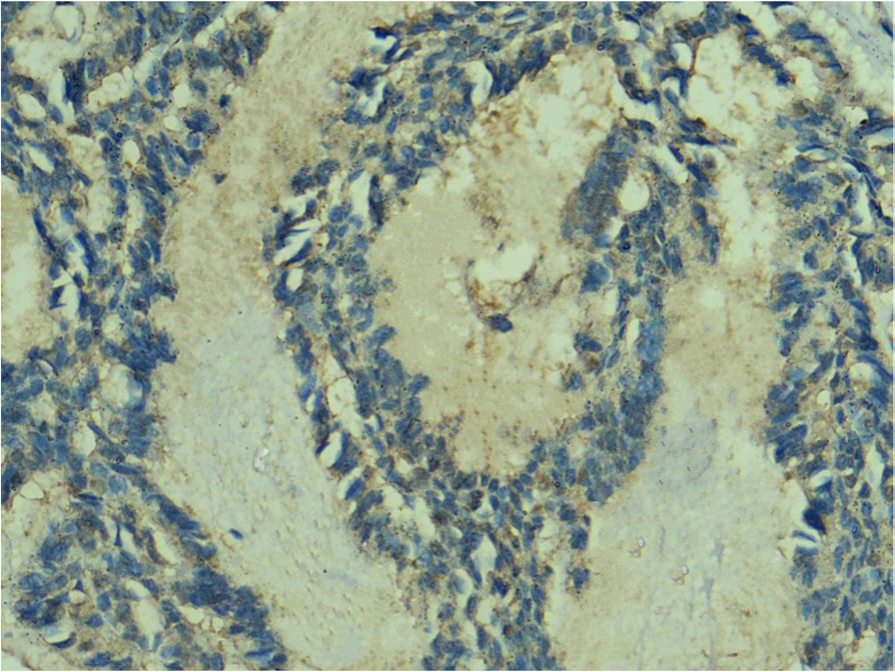
Photomicrograph showing negative HCV core antigen expression in a case of Canalicular Adenoma, magnification 400x

**Fig. 3:**
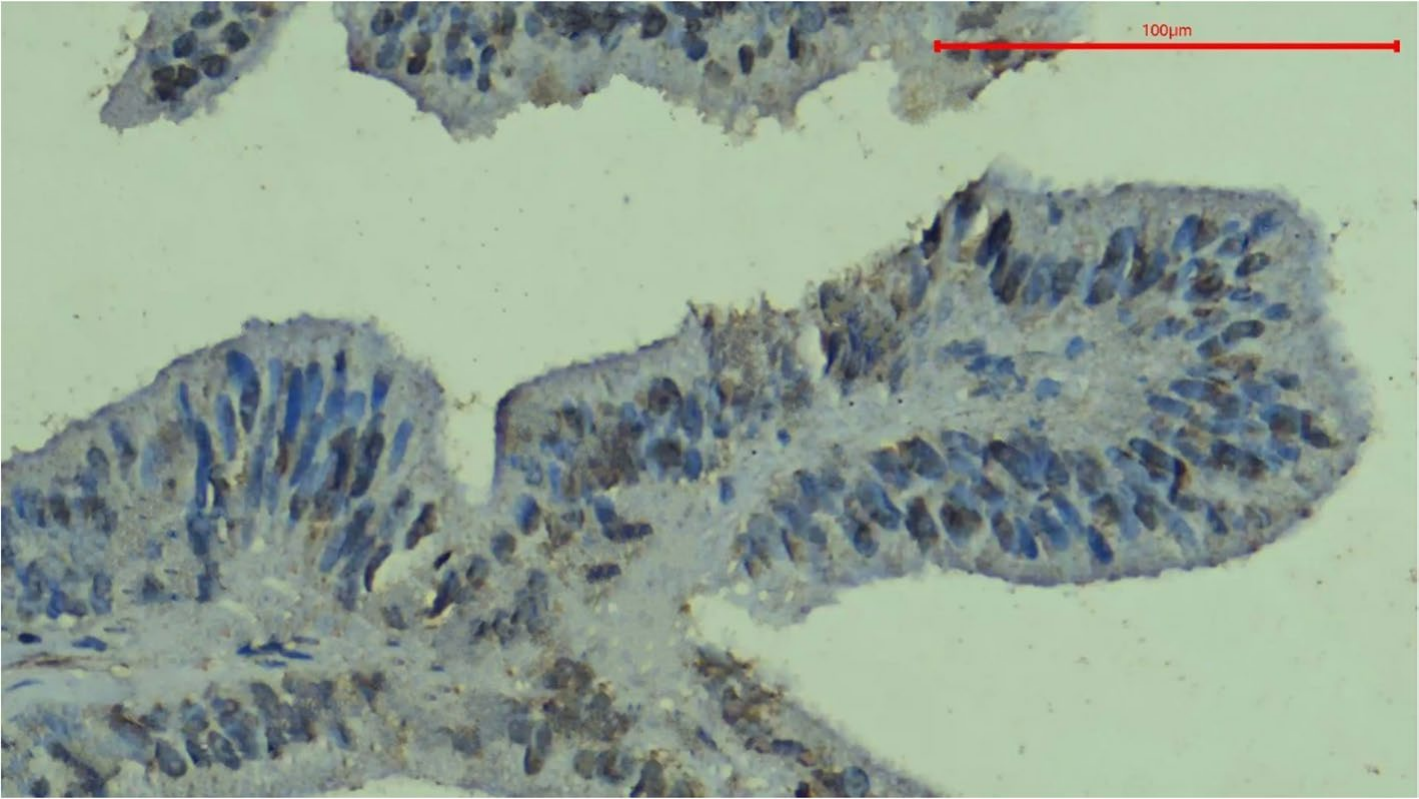
Photomicrograph showing negative HCV core antigen expression in a case of Papillary Oncocytic Cystadenoma, magnification 400x

**Fig. 4:**
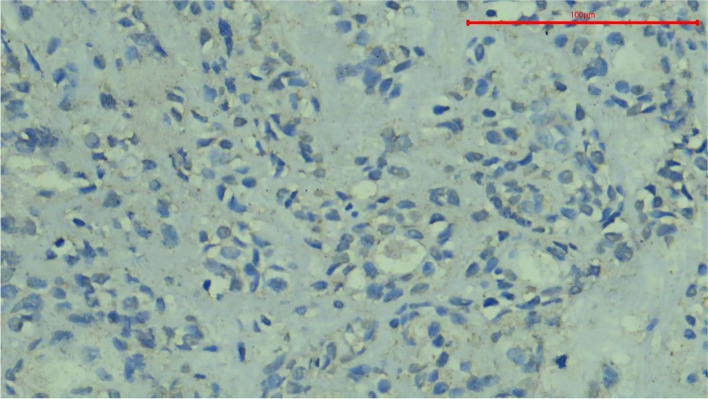
Photomicrograph showing negative HCV core antigen expression in a case of Pleomorphic Adenoma, magnification 400x

**Fig. 5:**
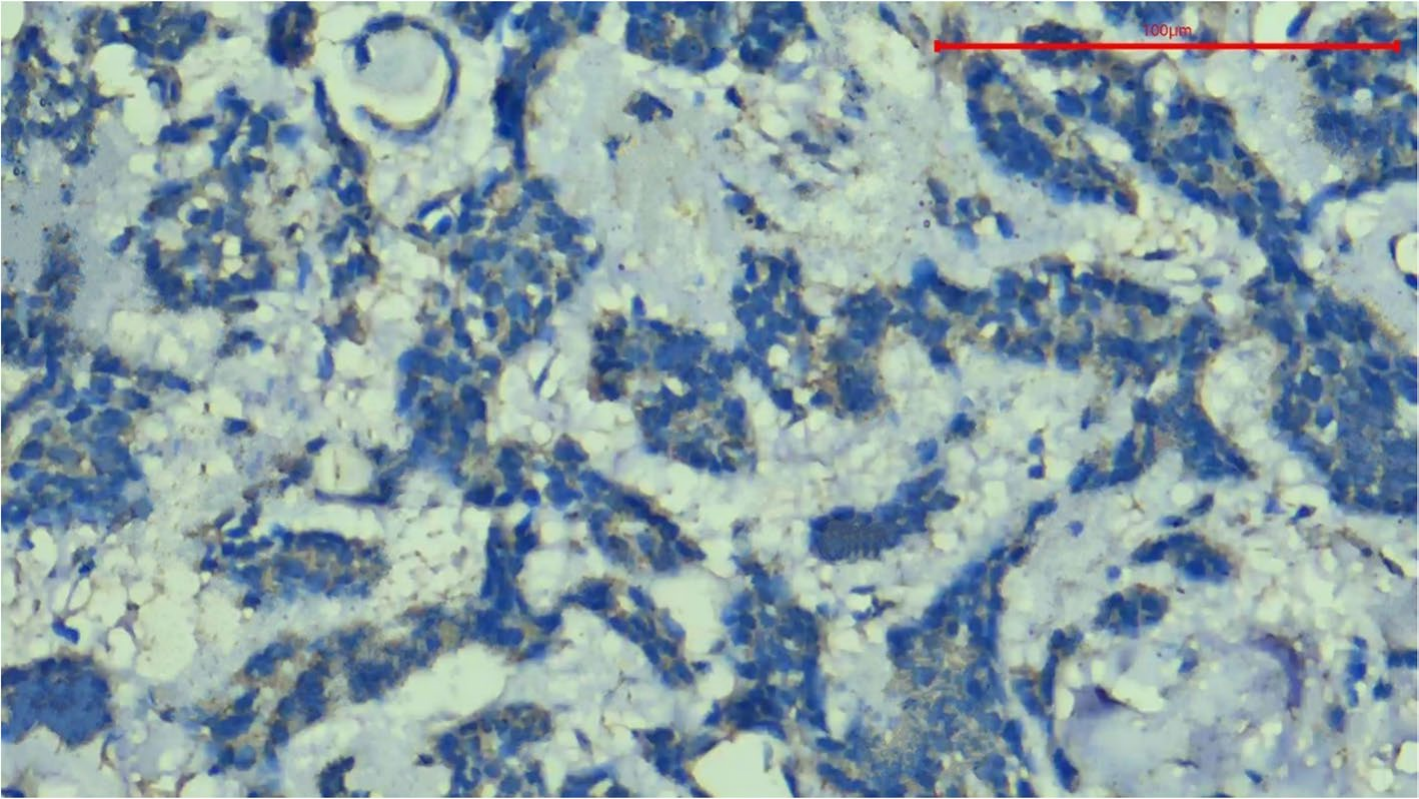
Photomicrograph showing negative HCV core antigen expression in a case of Adenoid Cystic Carcinoma, magnification 400x

Anti-HCV core antigen expression was absent in all the cases of Canalicular Adenoma (Fig. 2) as well as in cases of Papillary Oncocytic Cystadenoma (Fig. 3). Additionally, 6 out of the 21 Pleomorphic Adenomas showed negative expression (Fig. 4). When the expression of anti-HCV core antigen was examined in different types of malignant SGNs, Adenoid Cystic Carcinoma was the only case out of the 16 that showed negative expression (Fig. 5).

### Pattern of expression of HCV core antigen

**Fig. 6:**
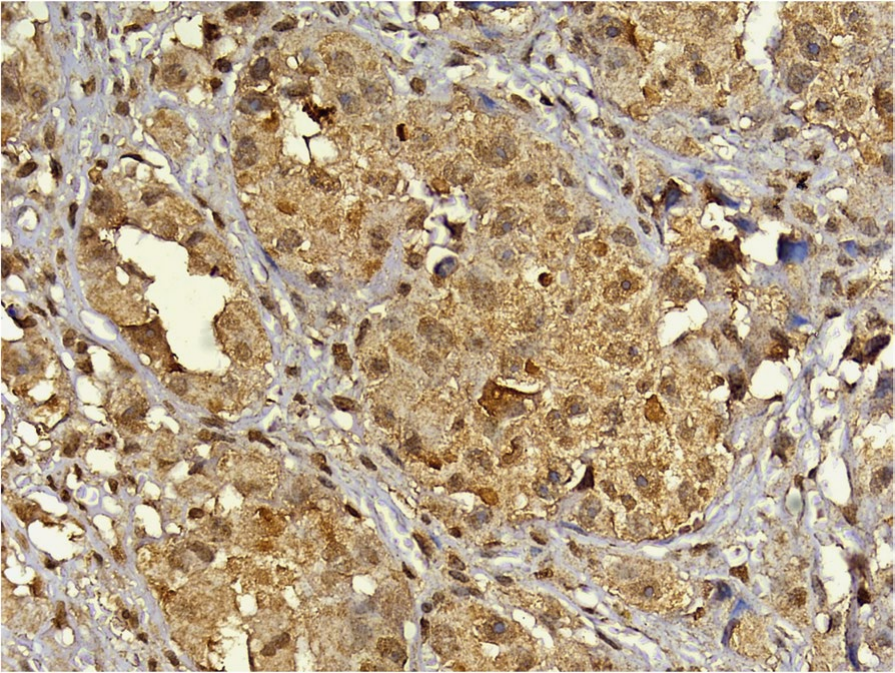
Photomicrograph of Oncocytic Carcinoma showing HCV core antigen immunopositivity. Note the diffuse granular cytoplasmic stain and nuclear expression with expression in fibroblasts and lymphocytes in the surrounding connective tissue, magnification 400x

To analyze the results, the location of expression was first evaluated within the tissues; the specific staining was granular. Staining was found exclusively in the cytoplasm of the tumor cells, with occasional instances of nuclear staining. Positive expression was also observed in some fibroblasts and lymphocytes in the surrounding connective tissue (Fig. 6). The staining pattern of the HCV core antigen by immunohistochemistry can exhibit various characteristics, reflecting the intracellular distribution of the viral protein. These staining patterns include diffuse cytoplasmic, patchy cytoplasmic, nuclear, or a combination of both the nucleus and cytoplasm.

**Fig. 7:**
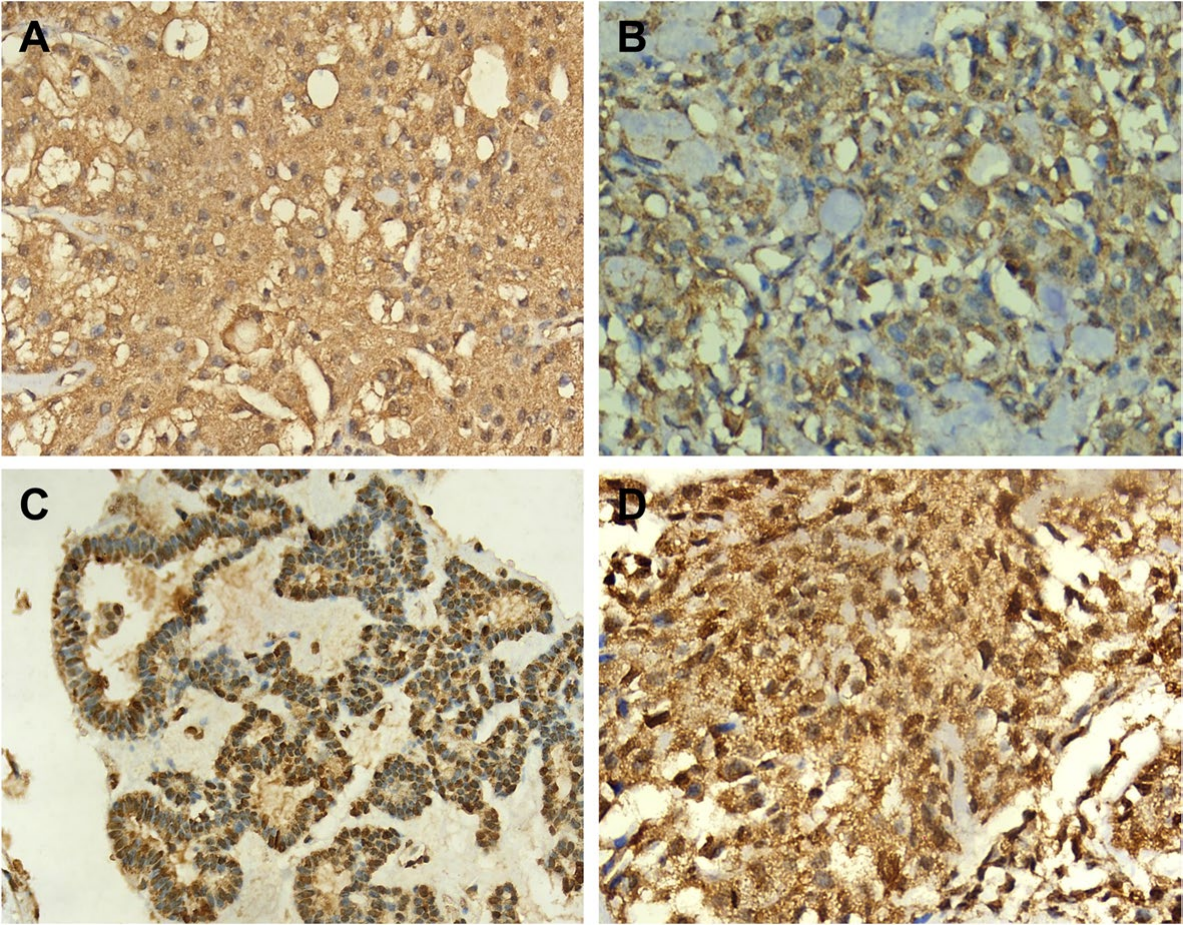
Photomicrograph of Mucoepidermoid Carcinoma showing HCV core antigen immunopositivity. The stain exhibits a diffuse granular cytoplasmic pattern with no nuclear expression, magnification 400x (**A**). Photomicrograph of Pleomorphic Adenoma showing HCV core antigen immunopositivity. The stain shows a patchy and granular cytoplasmic pattern and absence of nuclear expression, magnification 400x (**B**). Photomicrograph of Basal Cell Adenoma showing nuclear expression of HCV core antigen. Note the peripheral palisading of the nuclei, magnification 400x (**C**). Photomicrograph of another case of Pleomorphic Adenoma showing both nuclear and cytoplasmic HCV core antigen immunopositivity. Note the granular cytoplasmic stain, magnification 400x (**D**)

The diffuse cytoplasmic pattern indicated uniform distribution of the antigen throughout the cytoplasm of the infected cells (Fig. 7A). Patchy cytoplasmic staining signifies sporadic distribution of the antigen, resulting in irregularly scattered positive areas within the cytoplasm (Fig. 7B). The nuclear staining pattern indicated the presence of the HCV core antigen within the cell nuclei (Fig. 7C). Additionally, it was possible to observe a combination of staining patterns, where both cytoplasmic and nuclear staining coexisted within the same cell (Fig. 7D).

### Comparison between benign and malignant tumors

There was no statistically significant difference between the expression patterns of benign and malignant tumors (*P*-value = 0.616, Effect size = 0.285) (Table 4).

**Table 4:** Frequencies (n), percentages (%), and results of Fisher’s Exact test for comparison between expression patterns in benign and malignant tumors

Expression pattern	Benign (*n* = 18)		Malignant (*n* = 15)		*P*-value	*Effect size (v)*
	n	%	n	%		
Diffuse cytoplasmic	5	27.8	3	20	0.616	0.285
Patchy cytoplasmic	3	16.7	2	13.3		
Nuclear	2	11.1	0	0		
Both	8	44.4	10	66.7		

^*^Significant at *P* ≤ 0.05

### Benign salivary gland neoplasms

#### Pleomorphic adenoma

Among the 15 positive Pleomorphic Adenoma cases, 8 out of these 15 exhibited dual expression, 5 out of the 15 displayed diffuse cytoplasmic staining, and the remaining two demonstrated patchy cytoplasmic staining.

#### Basal cell adenoma

Of the three positive Basal Cell Adenoma cases, two specifically exhibited nuclear expression, whereas the remaining case displayed patchy cytoplasmic staining.

#### Comparison between expression patterns of pleomorphic adenoma and other benign tumors

A statistically significant difference was observed between the expression patterns of Pleomorphic Adenoma and other benign tumors (*P*-value = 0.005, effect size = 0.856) (Table 5). Pleomorphic Adenoma cases showed a lower prevalence of patchy cytoplasmic and nuclear expression, and a higher prevalence of diffuse cytoplasmic and both expression patterns than other benign tumors.

**Table 5:** Frequencies (n), percentages (%), and results of Fisher’s Exact test for comparison between the expression patterns of Pleomorphic Adenoma and other benign tumors

Expression pattern	Pleomorphic Adenoma		Other benign tumors		*P*-value	*Effect size (v)*
	n	%	n	%		
Diffuse cytoplasmic	5	33.3	0	0	0.005*	0.856
Patchy cytoplasmic	2	13.3	1	33.3		
Nuclear	0	0	2	66.7		
Both	8	53.3	0	0		

^*^Significant at *P* ≤ 0.05

### Malignant salivary gland neoplasms

#### Mucoepidermoid Carcinoma

Among the 10 Mucoepidermoid Carcinoma cases that were positive, 2 were classified as low-grade, while the rest were categorized as high-grade. Of these 10 cases, 8 showed dual expression, 1 displayed diffuse cytoplasmic staining, and the remaining case exhibited patchy cytoplasmic staining.

#### Myoepithelial Carcinoma

When examining the expression of anti-HCV core antigen within Myoepithelial Carcinoma, it was discovered that the two cases exhibited dual expression of the anti-HCV core antigen in the nucleus and cytoplasm.

#### Oncocytic Carcinoma

When examining the expression of anti-HCV core antigen within Oncocytic Carcinoma, it was found that among the two positive cases, one demonstrated dual expression of the anti-HCV core antigen in the nucleus and cytoplasm, while the other displayed patchy cytoplasmic staining.

#### Adenoid Cystic Carcinoma

When examining the expression of anti-HCV core antigen within Adenoid Cystic Carcinoma, it was discovered that the only positive case exhibited dual expression of the anti-HCV core antigen.

#### Comparison between expression patterns of Mucoepidermoid Carcinoma and other malignant tumors

There was no statistically significant difference between the expression patterns in Mucoepidermoid Carcinoma and other malignant tumors (*P*-value = 0.550, effect size = 0.361) (Table 6).

**Table 6:** Frequencies (n), percentages (%), and results of Fisher’s Exact test for comparison between the expression patterns of Mucoepidermoid Carcinoma and other malignant tumors

Expression pattern	Mucoepidermoid Carcinoma		Other malignant tumors		*P*-value	*Effect size (v)*
	n	%	n	%		
Diffuse cytoplasmic	3	30	0	0	0.550	0.361
Patchy cytoplasmic	1	10	1	20		
Nuclear	0	0	0	0		
Both nuclear and cytoplasmic	6	60	4	80		

^*^Significant at *P* ≤ 0.05

### Immunoexpression by area percentage

#### Comparison between benign and malignant tumors

**Fig. 8:**
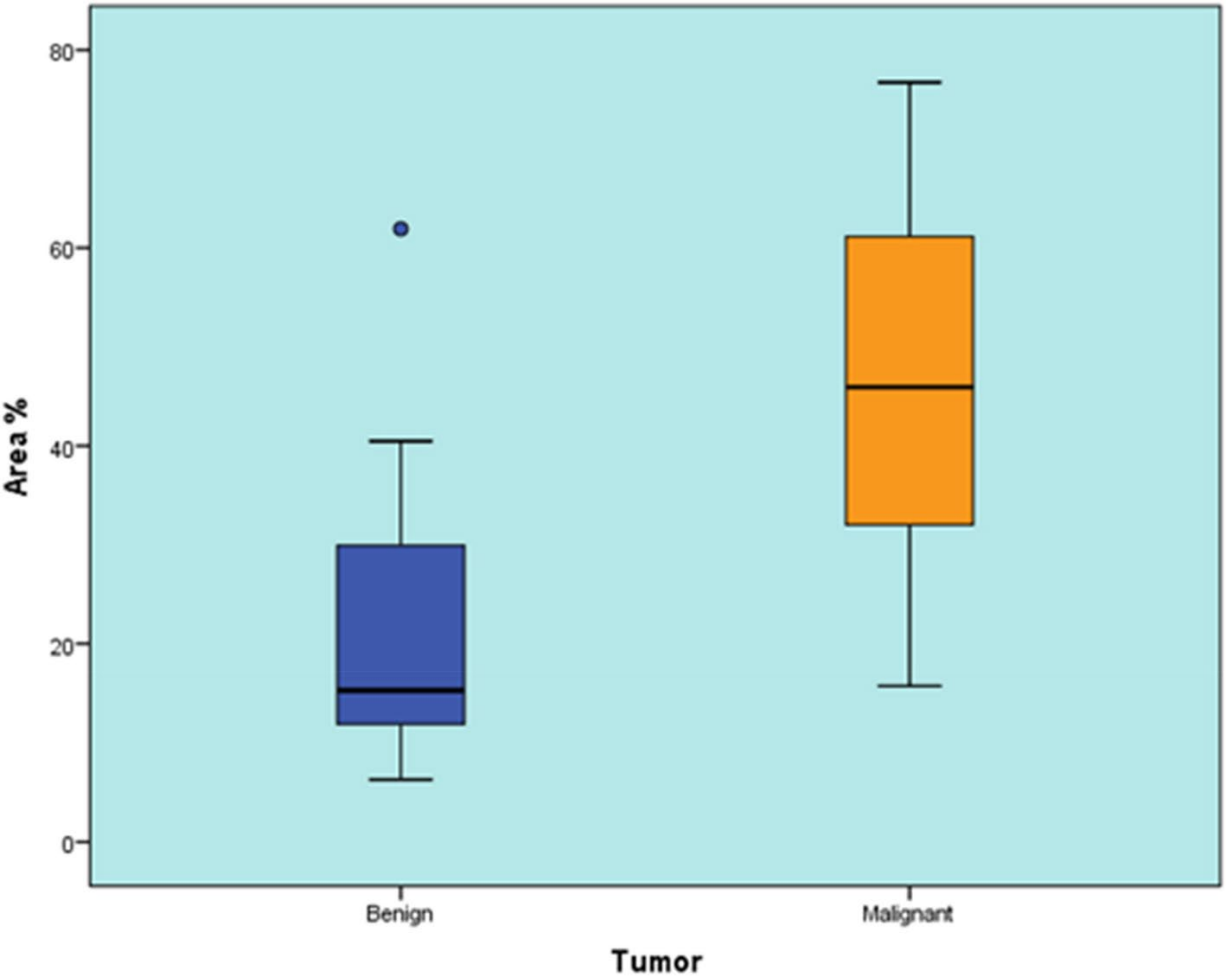
Box plot representing median and range values for area % in benign and malignant tumors (Circle represent outlier)

Benign tumors showed a statistically significant lower area percentage than malignant tumors (*P*-value < 0.001, Effect size = 1.791) (Table 7) (Fig. 8).

**Table 7:** Descriptive statistics and results of Mann-Whitney U test for comparison between area % in benign and malignant tumors

Benign (*n* = 28)		Malignant (*n* = 16)		*P*-value	*Effect size (d)*
Median (Range)	Mean (SD)	Median (Range)	Mean (SD)		
15.28 (6.29-61.92)	20.94 (14.38)	45.92 (15.74-76.74)	48.02 (17.79)	< 0.001*	1.791

^*^Significant at *P* ≤ 0.05

#### Comparison between area percentage of Pleomorphic Adenoma and other benign tumors

**Fig. 9:**
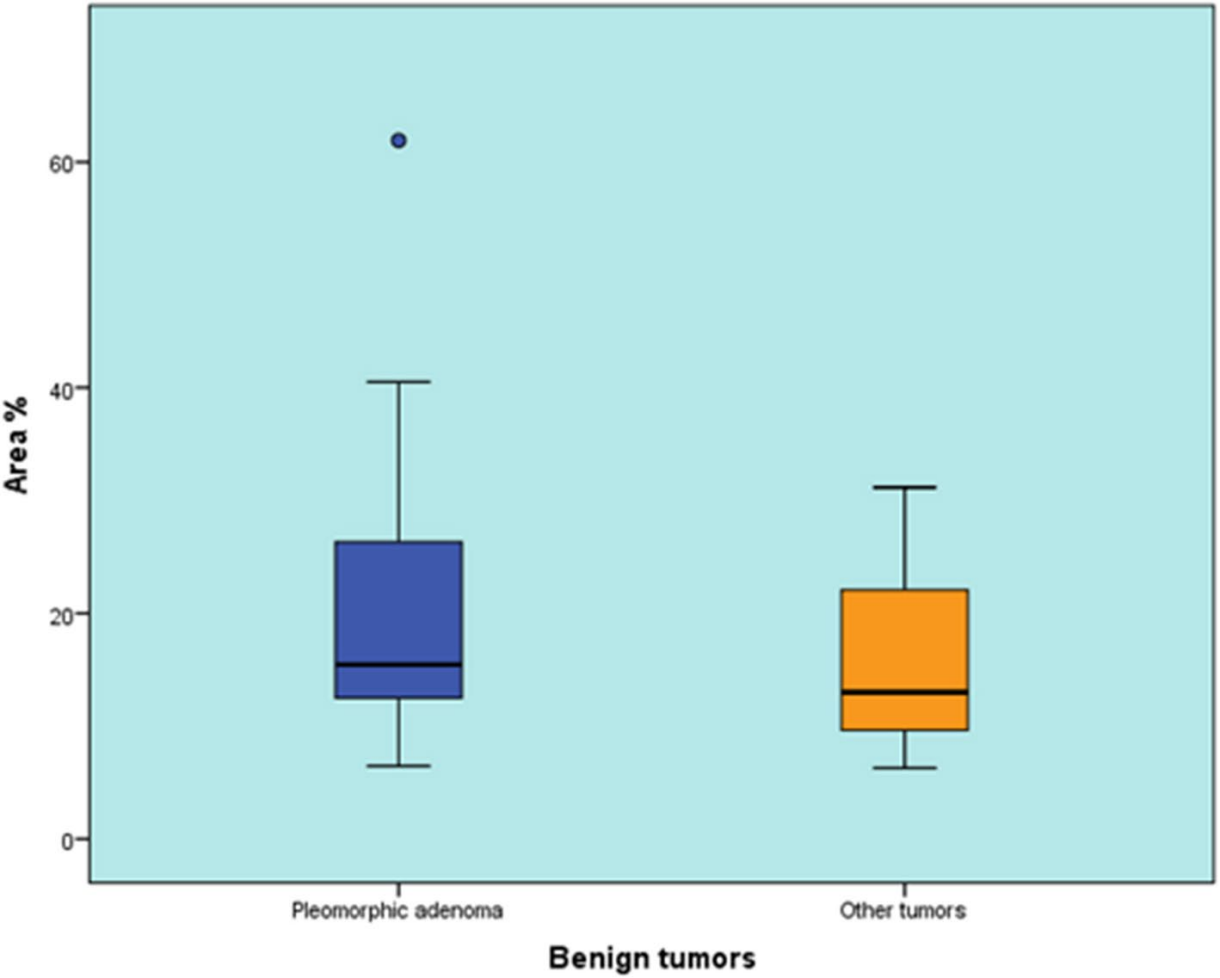
Box plot representing median and range values for area % of Pleomorphic Adenoma and other benign tumors (Circle represent outlier)

There was no statistically significant difference between the area percentage of Pleomorphic Adenoma and other benign tumors (*P*-value = 0.441, Effect size = 0.369) (Table 8) (Fig. 9).

**Table 8:** Descriptive statistics and results of Mann-Whitney U test for comparison between area % of Pleomorphic Adenoma and other benign tumors

Pleomorphic adenoma		Other benign tumors		*P*-value	*Effect size (d)*
Median (Range)	Mean (SD)	Median (Range)	Mean (SD)		
15.46 (6.46-61.92)	21.77 (14.94)	13.01 (6.29-31.14)	16.81 (12.85)	0.441	0.369

^*^Significant at *P* ≤ 0.05

### Comparison between area percentage of Mucoepidermoid Carcinoma and other malignant tumors

**Fig. 10:**
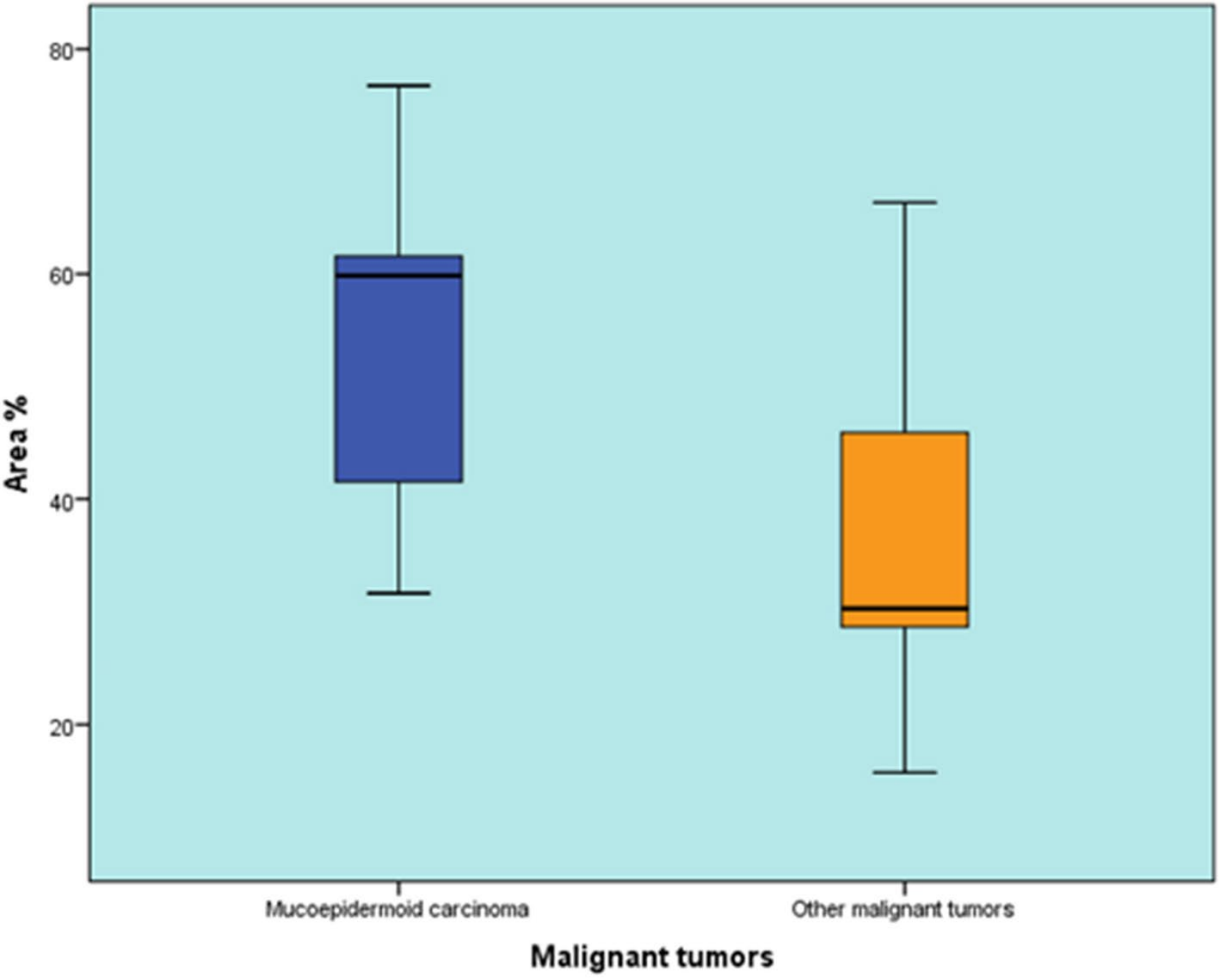
Box plot representing median and range values for area % of Mucoepidermoid Carcinoma and other malignant tumors

There was no statistically significant difference between the area percentage of Mucoepidermoid Carcinoma and other malignant tumors (*P*-value = 0.142, Effect size = 0.82) (Table 9) (Fig. 10).

**Table 9:** Descriptive statistics and results of Mann-Whitney U test for comparison between area % of Mucoepidermoid Carcinoma and other malignant tumors

Mucoepidermoid Carcinoma		Other malignant tumors		*P*-value	*Effect size (d)*
Median (Range)	Mean (SD)	Median (Range)	Mean (SD)		
59.85 (31.64-76.74)	53.32 (15.2)	30.3 (15.74-66.36)	37.4 (19.41)	0.142	0.82

^*^Significant at *P* ≤ 0.05

### Detection of HCV RNA by reverse transcription polymerase chain reaction

**Fig. 11:**
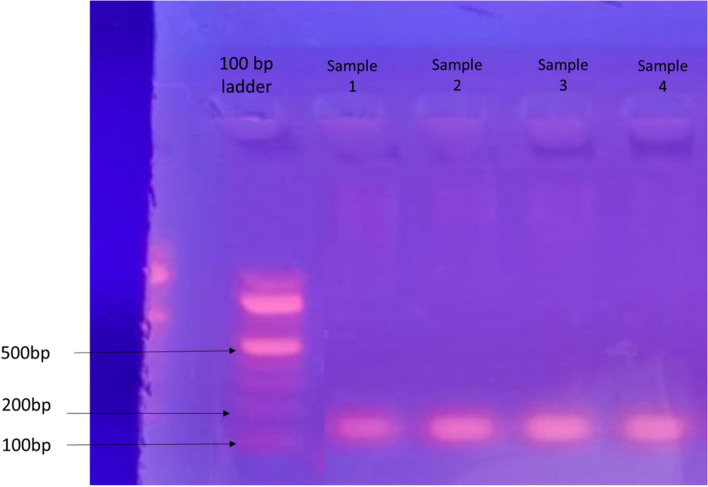
The agarose gel electrophoresis revealed the presence of a PCR-amplified product of HCV RNA with an estimated size of 100 base pairs

To verify the presence of HCV RNA, a confirmatory RT-PCR analysis was conducted. Given that PA and MEC were the predominant tumor types in this study, a total of four cases were chosen—two representing each tumor type. In the case of MEC, one high-grade and one low-grade case were specifically selected. The results showed the detection of HCV RNA in all four samples at a size of 100 base pairs (Fig. 11).

The choice of PA and MEC as the selected tumor types was based on their prevalence within their respective categories. The selection of the four cases was based on the availability of sufficient tissue in the archival block that is necessary for PCR analysis.

## Discussion

Chronic HCV infection extends beyond liver-related complications, manifesting as diverse systemic illnesses and extrahepatic manifestations (EHMs) [13]. At the national level, Egypt has faced a significant HCV epidemic, with substantial endemic transmission. The prevalence of HCV is elevated across all population groups, indicating the widespread nature of this epidemic, which is not restricted to specific regions or segments of the population [14].

For years, Egypt had the highest incidence of HCV infection globally. However, the country has made remarkable progress in limiting the spread of this disease through extensive diagnostic and treatment campaigns. These campaigns employ highly effective regimens of direct-acting antiviral therapy, resulting in the mass treatment of almost 2.5 million patients with HCV [7]. Regardless of these efforts, there remains a possibility that the cumulative effects of viral damage on tissues may have already occurred.

In addition to the liver, chronic HCV infection results in diverse systemic illnesses. Some EHMs are immune-mediated, while others appear to be caused by chronic inflammation as well as complex oral manifestations. Clinicians must be well-informed about these EHMs, as they can significantly impact the care of patients with HCV infection [15]. A substantial proportion of individuals with chronic HCV infection experience at least one clinical manifestation outside the liver, including sialadenitis, chronic enlargement of the major salivary glands, and the commonly reported symptoms of xerostomia and sicca syndrome (resembling Sjogren syndrome) [16].

Despite research, the etiology of SGNs remains unclear [17]. Risk factors include aging, radiation, chemicals, and exposure to viruses [18]. Understanding the connection between viruses and cancer is crucial for the development of strategies to prevent, diagnose, and treat virus-related cancers [19].

To date, no study has examined the link between HCV and SGNs in the Egyptian population. Therefore, this study is the first to be carried out in an attempt to detect the possible association between HCV and epithelial SGNs.

The detection of HCV antigens using immunohistochemistry has the potential to provide valuable pathological information. This could allow the establishment of a correlation between the sites of viral replication and tissue damage [20]. Immunohistochemistry has been used in multiple studies to detect HCV antigens in FFPE tissue sections. Based on the results of previous studies that employed immunohistochemistry to detect HCV antigens, the same methodology was utilized to identify HCV core antigen in SGNs in this study. One study investigating the expression of HCVNS3 antibody in potentially malignant disorders (PMDs) and Oral Squamous Cell Carcinoma (OSCC) through immunohistochemistry found positive HCVNS3 expression in a subset of cases, suggesting the potential involvement of HCV in PMDs and OSCC development, with HCV co-infection correlating with poorer survival outcomes in OSCC [21].

Similarly, in another recent study, researchers examined HCV core antigen using immunohistochemistry to investigate the possible association between HCV and OSCC. They found a relatively high frequency of HCV among OSCC cases in Egypt. Interestingly, the highest incidence of HCV infection was observed in cases of moderately differentiated OSCC. Furthermore, poorly differentiated OSCC showed a higher cell count, suggesting a potential correlation between increased viral load and more aggressive tumor behavior [22].

The interpretation of immunohistochemical staining patterns for HCV core antigen is crucial for understanding the localization and distribution of the viral protein within the tissue sample. Such observations will aid in the diagnosis and study of HCV infection, allowing researchers and clinicians to gain valuable insights into the pathogenesis and behavior of the virus within the host.

Cells that exhibited staining in the cytoplasm, nucleus, or both were considered to be positive. Heterogeneity in staining patterns may be linked to the viral load or replication level, affecting the availability of viral particles for detection [23]. The staining patterns observed in this study included patchy cytoplasmic, diffuse cytoplasmic, and nuclear staining. Interestingly, a combination of these patterns was found in a significant percentage of both benign and malignant positive cases. This finding aligns with the results of Shiha et al. (2005), who also noted a comparable pattern of HCV core antigen expression in FFPE liver tissue [23]. However, there was no statistically significant difference in expression patterns between benign and malignant tumors (*P*-value = 0.090).

The subcellular localization of HCV core protein is a topic of ongoing research and debate. Initially released from the viral polyprotein within the endoplasmic reticulum (ER) membrane, the core protein undergoes further processing and is localized primarily to the ER, mitochondria, and lipid droplets. However, uncertainties persist regarding the specific sequences responsible for targeting the core protein to these organelles [24]. Its association with the mitochondrial membrane suggests its potential role in modulating mitochondrial function, affecting cellular processes such as gene expression, proliferation, and apoptosis [25].

Moreover, the presence of nuclear localization signals (NLS) in the HCV core protein indicates its ability to translocate to the nucleus. NLS motifs, which contain basic amino acids such as lysine and arginine, serve as recognition signals for importin proteins involved in nuclear import. In the cytoplasm, these motifs interact with importin proteins, particularly importin-α, which forms a complex with importin-β to facilitate its nuclear translocation through nuclear pore complexes. However, conflicting reports and variations in experimental conditions have contributed to the ongoing debate surrounding the precise subcellular localization of the HCV core protein [24].

A previous study suggested that the nuclear localization of the core protein is associated with its oncogenic activity and may contribute to liver disease progression. While HCV core is predominantly found in the cytoplasm of liver biopsy samples from HCV-infected patients, with only occasional detection in the nucleus of infected hepatocytes, truncated core proteins have been observed in the nucleus of tumor tissues from HCV-related hepatocarcinoma patients. Furthermore, this study highlights the importance of understanding the mechanisms regulating the subcellular distribution and trafficking of the HCV core protein, considering its role as a major viral factor in liver disease and HCC [26, 27]. The nuclear localization of HCV core antigen in samples of malignant SGNs observed in this study can potentially be explained by this phenomenon.

In this study, an interesting observation was made that two cases of Basal Cell Adenoma exhibited nuclear expression for HCV core antigen. Although Basal Cell Adenoma is classified as a benign tumor, the presence of nuclear expression of HCV core antigen raises concerns regarding its potential significance. Consequently, regular follow-up periods are warranted to monitor progression from Basal Cell Adenoma to Basal Cell Adenocarcinoma. This will enable early intervention and appropriate management if the tumor progresses to a more aggressive form.

Claudin 1 (CLDN1) is a tight junction protein that has been extensively studied in the context of HCV infection. It plays a crucial role in the virus's entry into hepatocytes by facilitating viral attachment and engagement with coreceptors. High expression of CLDN1 in hepatocytes makes them susceptible to HCV infection [28]. Interestingly, similar expression of CLDN1 has been observed in both major and minor salivary gland ducts, suggesting its potential involvement in HCV infection outside the liver [29]. This finding may explain the prominent expression of HCV core protein in PA, which is an intercalated duct-derived tumor.

In a recent study, the expression of claudin proteins (CLDN-1, -3, -4, -5, and -7) was examined in salivary gland MEC. Previous research has suggested that claudin deregulation could contribute to cancer development by disrupting cell-cell adhesion and polarity. Contrary to expectations, this study found high and widespread expression of these claudin proteins in MEC, regardless of tumor grading, predominantly on the cell membrane surrounding intermediate and epidermoid cells. This finding contradicts the notion that loss of claudin expression is associated with carcinogenesis and may offer insights into the positive expression of HCV core protein observed in MEC cases in this study [30]. However, further research is needed to fully understand the significance of CLDN1 expression in non-hepatic tissues and its implications in HCV infection.

The measurement of the antigen-positive area revealed that the positive area percentage of PA did not exhibit a statistically significant difference when compared to other benign tumors (*P*-value = 0.441). Nevertheless, there was a statistically significant difference in expression patterns between PA and other benign tumors. PA demonstrated a higher occurrence of diffuse cytoplasmic and dual expression patterns compared to other benign tumors. Statistically, no significant difference was observed in the positive area percentage between MEC and other malignant tumors.

In contrast, a study conducted in 1997 investigated whether HCV infects epithelial cells of the salivary glands in patients with HCV. The study found no evidence of HCV RNA in salivary gland epithelial cells, leading to the conclusion that HCV does not infect these cells in patients with active viral infection [31].

In a similar study, researchers examined the prevalence of HCV RNA in saliva and salivary glands, and its potential association with salivary gland disorders in patients with chronic hepatitis C. However, the results did not show any significant association between the presence of HCV RNA in saliva or salivary glands and the occurrence of xerostomia, hyposalivation, or sialadenitis. These findings suggest that while HCV may not directly cause these salivary gland disorders, there could be an indirect involvement through immune-mediated mechanisms in their development among patients with chronic hepatitis C [8].

One of the limitations of this study was that employing a series of large cohorts might be more advantageous in establishing a potential association between HCV and SGNs. This approach requires a longer duration because of the relatively infrequent incidence of SGNs.

In conclusion, the findings of this study indicated a higher occurrence of HCV in SGNs, especially in cases of malignancy. This suggests a potential association, but further studies involving larger cohorts are necessary. Such investigations would highlight the significance of regular oral cavity examinations for individuals with HCV, possibly leading to early detection of neoplastic changes and thereby improving treatment outcomes.

Furthermore, this emphasizes the significance of including HCV screening as part of routine work-up in patients reporting unexplained xerostomia, salivary gland enlargement or neoplastic lesions. Such screening can help identify individuals who may be unknowingly harboring the virus, serving as a preventive measure to reduce its transmission. Additionally, confirming this correlation will draw greater attention to the imperative of conducting further research to explain the exact mechanisms underlying this association.

## Data Availability

The data that support the findings of this study are available from the corresponding author upon request.

## Abbreviations

SGNs: Salivary gland neoplasms
HCV: Hepatitis C
WHO: World Health Organization
FFPE: Formalin-fixed paraffin-embedded
RT-PCR: Reverse transcription polymerase chain reaction
EHMs: Extrahepatic manifestations
PMDs: Potentially malignant disorders
OSCC: Oral Squamous Cell Carcinoma
PA: Pleomorphic Adeonoma
MEC: Mucoepidermoid Carcinoma
ER: Endoplasmic reticulum
NLS: Nuclear localization signals
CLDN1: Claudin 1
